# Lingual Fasciculation as a Point of Call for the Diagnosis of Amyotrophic Lateral Sclerosis: A Literature Review

**DOI:** 10.7759/cureus.64153

**Published:** 2024-07-09

**Authors:** leon Galeazzi, Judith Holzman, André Porporatti, Juliette Rochefort

**Affiliations:** 1 Oral Surgery, Pitie Salpetriere Hospital, AP-HP (Assistance Publique – Hôpitaux de Paris) Paris Cité University, Paris, FRA; 2 Dentistry, Pitie Salpetriere Hospital, AP-HP (Assistance Publique – Hôpitaux de Paris) Paris Cité University, Paris, FRA; 3 Dentistry, Paris Cité University, Paris, FRA

**Keywords:** amyotrophic lateral sclerosis – frontotemporal spectrum disorder, oral diseases, lingual disease [mesh terms], tongue disease [mesh terms], fasciculation [mesh terms]

## Abstract

Background and aim: Dental surgeons often play a pivotal role in the initial detection of lingual fasciculations (LFs). These involuntary micro-movements of the tongue can serve as early clinical indicators of neurodegenerative diseases, with amyotrophic lateral sclerosis (ALS) being the most concerning. Therefore, it is imperative to educate dental surgeons on identifying LF and understanding the potential underlying pathologies.

Objectives: This study aimed to pinpoint the pathologies in which LFs could emerge as an early clinical marker. Our review focused on articles delineating patient populations exhibiting LF within broader pathological contexts, encompassing neurological and other conditions, with the aim of elucidating their etiologies.

Methods: We conducted a comprehensive literature review across four databases (PubMed, Embase, Web of Science, and Scopus). Two authors independently extracted data, with consultation from a third author when necessary. Eligible articles included those describing patients with LFs, detailing the methods of detection, diagnosis, and associated pathologies.

Results: Our review identified 22 articles encompassing 153 patients with LF, with an average age of 45.8 years and a female prevalence of 43%. Electromyography and ultrasound emerged as the predominant detection methods. ALS constituted the primary diagnosis in the majority of cases (91%). Additionally, other conditions diagnosed included Machado-Joseph disease (0.046%), familial transthyretin amyloid neuropathy (0.013%), Brown-Vialetto-Van-Laere syndrome (0.006%), chronic inflammatory demyelinating polyneuropathy (0.006%), bulbospinal amyotrophy or Kennedy's disease (0.006%), and osmotic demyelination syndrome (0.006%). LF secondary to organophosphate poisoning was also documented. Symptoms associated with LF encompassed taste alterations, dysphagia, difficulty swallowing, and slurred speech.

Conclusion: While primarily indicative of ALS, LFs may also signal diverse underlying pathologies. Healthcare practitioners should be vigilant in their detection and expedite patient referrals to facilitate early integration into care protocols.

## Introduction and background

Amyotrophic lateral sclerosis (ALS) stands as a devastating progressive neurodegenerative ailment that ravages nerve cells within the brain and spinal cord. Individuals afflicted by ALS endure a gradual severance of communication channels between muscles and the brain, culminating in the gradual loss of mobility, speech, feeding capabilities, and ultimately, respiratory function [1]. This affliction typically strikes individuals aged between 40 and 70 years, with over 5000 new cases diagnosed annually as of 2020. ALS can manifest in two forms: sporadic, accounting for 90% of cases, or familial, comprising the remaining 10% [2]. It originates either in the spinal or bulbar regions, with a bleak prognosis marked by an average survival span of merely 2-5 years. Early detection is paramount, offering the potential for enhanced disease management and improved quality of life throughout its progression. Regrettably, no definitive cure exists, with respiratory failure ultimately claiming the lives of eight out of 10 patients [1,2].

Other pathologies can be revealed by lingual fasciculations (LFs), such as Brown-Vialetto-Van Laere syndrome, chronic inflammatory demyelinating polyneuropathy, Machado-Joseph disease, Kennedy's disease, transthyretin familial amyloid polyneuropathy, and osmotic demyelination syndrome.

One of the key early indicators of these pathologies is the presence of LFs, which can be discerned by dentists and oral surgeons, often positioning them at the forefront of ALS diagnostic endeavors. Presently, two techniques serve in fasciculation detection: electromyogram (EMG) and muscle ultrasonography (MUS). While EMG remains the gold standard, it is accompanied by discomfort and duration, unlike the painless and wider-ranging exploration facilitated by MUS [3,4].

LFs manifest as involuntary, uncontrollable, and persistent movements of the tongue. They may arise suddenly, instigating sensations of oral dryness, speech impediments, and swallowing difficulties, prompting individuals to seek consultation with their dentist or primary care provider [3,5]. However, these disturbances may also herald various underlying pathologies. To facilitate prompt referral to the appropriate specialists, primarily neurologists, understanding their clinical features, detection and diagnostic methods, and associated conditions is imperative. This is the aim of the work presented here.

## Review

Materials and methods

Registration/Any Guideline Used

This literature review adheres to the guidelines outlined in the Joanna Briggs Institute guide [6]. The methodology employed in this literature review aligns with the Preferred Reporting Items for Systematic Reviews and Meta-Analyses (PRISMA) requirements. The primary research question guiding this review was: "How to detect and diagnose lingual fasciculations and what pathologies could be associated?" The research protocol for this review was registered with the International Prospective Register of Systematic Reviews (PROSPERO) under registration number CRD42024540809.

Objectives/PCC Framework/Definition of Variables

The primary aim of this study was to delineate the characteristics of LFs and establish methodologies for their detection and diagnosis.

Additionally, the secondary objective was to ascertain the pathologies in which LFs could serve as an early clinical indicator. Our review specifically targeted articles detailing patient cohorts displaying LFs (Population) amidst a broader spectrum of pathologies (Context), encompassing neurological and other conditions (Concept). The overarching goal was to compile a comprehensive list of their etiologies.

Inclusion Criteria

To be considered for inclusion, articles were required to delineate the distinct characteristics of LFs. Additionally, we included articles that comprehensively detailed all pathologies associated with LFs.

Exclusion Criteria

Duplicates within the same database were eliminated, followed by the incorporation of searches across various databases using bibliographic reference management software. Articles deemed unrelated to the topic were also excluded from consideration.

Search Strategy

We conducted searches across several databases including MEDLINE/PubMed, Scopus, Web of Science, and Embase. Additionally, we explored gray literature sources such as OpenGrey and Google Scholar. The search terms utilized were ("tongue" OR "tongues" OR "Tongue Diseases" OR "lingual") AND ("fasciculate" OR "fasciculated" OR "fasciculates" OR "fasciculating" OR "fasciculation" OR "fasciculations" OR "fascicule" OR "fascicules"). The specific search equations employed are documented in Table 1.

**Table 1:** The specific search equations employed for each database.

Database	Search equations employed
PubMed	“(Tongue OR tongues OR "tongue" [MeSH Terms] OR "Tongue Diseases" [MeSH Terms] OR lingual) AND (fasciculate" [All Fields] OR "fasciculated" [All Fields] OR "fasciculates" [All Fields] OR "fasciculating" [All Fields] OR "fasciculation" [MeSH Terms] OR "fasciculations" [All Fields] OR "fascicule" [All Fields])”
Embase	“(tongue OR tongues OR lingual) AND (fasciculate OR fasciculated OR fasciculates OR fasciculating OR fasciculations OR fascicule OR fascicules)”
Scopus	“(tongue OR tongues OR lingual) AND (fasciculate OR fasciculated OR fasciculates OR fasciculating OR fasciculations OR fascicule OR fascicules)”
Web of Science	“(tongue OR tongues OR lingual) AND (fasciculate OR fasciculated OR fasciculates OR fasciculating OR fasciculation OR fasciculations OR fascicule OR fascicules).”

Studies were considered eligible for inclusion if they were published between January 1, 2012, and July 15, 2022. Language restrictions were waived if translations were available. No constraints were imposed regarding the study design. In instances where studies lacked an abstract or full text, the authors (LG and JH) reached out to the respective authors. If no response was received, the record was excluded from consideration.

Data Extraction

Data extraction was carried out independently by two authors (LG and JH), with consultation from a third author (JR) in cases of disagreement. Following the removal of duplicates, the titles and abstracts of articles retrieved from the databases were screened in accordance with the predetermined inclusion and exclusion criteria. Additionally, the bibliographic sources of selected articles were scrutinized to identify any additional relevant studies not captured by our initial search strategy. Authors (LG and JH) extracted pertinent details pertaining to study design, participant demographics, interventions, comparators, and outcomes.

For each article, the following information was recorded: author(s), year of publication, the number of patients, their gender, average age at the time of LF diagnosis, and associated pathologies. These are documented and summarized in Table 2. The statistical analyses were performed using Microsoft Excel software.

**Table 2:** Clinical cases reported ALS: amyotrophic lateral sclerosis; BVVL: Brown-Van Vialetto-Van Laere syndrome; CIDP: chronic inflammatory demyelinating polyradiculoneuritis; SBMA: Kennedy's disease (bulbospinal muscular atrophy); ASC3: Machado-Joseph's disease (spinocerebellar ataxia type 3); TTR-FAP: familial amyloid polyneuropathy; n, number of cases reported per article; Cp. organoP, organophosphorus compounds; F: fasciculations; LF: lingual fasciculations.

Authors	n=	Sex	Age	Disease	Clinical signs
Vishnu et al. 2014 [7]	1	M	36	SLA	Dysphagia and LF
Kranthi et al. 2020 [8]	1	M	16	BVVL	Atrophied tongue with LF
Roberto et al. 2020 [9]	1	M	31	CIDP	Bilateral facial paresis and LF
Nusrat et al. 2019 [10]	1	M	28	SLA	LF, dysphonia, dysphagia
Rezende Filho et al. 2019 [11]	7	F: 56%, M: 44%	46.2	ASC3	LF and involuntary tongue closure/opening
Ranjan et al. 2019 [12]	1	F	19	Cp. organoP	Perioral F, FL
Suzuki et al. 2022 [3]	51	F: 51%, M: 49%	66.9	SLA	LF
Pelletier et al. 2013 [13]	7	F: 60%, M: 40%	58.8	SLA	LF and taste alteration
Martin et al. 2020 [14]	4	F: 42%, M: 58%	53.2	SLA	LF
Juan et al. 2020 [15]	3	F: 30%, M: 70%	62.7	SLA	LF
Araki et al. 2015 [16]	1	M	33	SBMA	LF
Pan et al. 2016 [17]	62	F: 41%, M: 59%	52	SLA	LF and fibrillations
Tsunoda et al. 2019 [18]	4	/	/	SLA	LF
Goyal and Mozaffar 2015 [19]	2	M: 100%	1: 75, 2: 60	TTR-FAP	Atrophy and LF
Herath et al. 2018 [20]	1	M	32	Alcoholism	LF, dysphagia, dysphonia, excessive salivation
Hagiwara et al. 2021 [21]	6	F: 34%, M: 66%	63.7	SLA	LF

Quality Assessment

Authors (LG and JR) have classified the levels of evidence of the obtained articles (Table 3) using guidelines from the HAS literature analysis guide (Haute Autorité de Santé. Niveau de preuve et gradation des recommandations de bonne pratique, 2013) and the Oxford levels of evidence [22].

**Table 3:** Level of evidence of the articles Analyzed according to HAS literature analysis guide and the Oxford levels of evidence.

Authors	Date	Article type	OMS grade	Oxford
Vishnu et al. [7]	2014	Case Report	Grade C Level 4	Step 4 Level 4
Kranthi et al. [8]	2020	Case Report	Grade C Level 4	Step 4 Level 4
Roberto et al. [9]	2020	Case Report	Grade C Level 4	Step 4 Level 4
Tsuji et al. [23]	2020	Prospective Cohort Study	Grade B Level 2	Step 3 Level 3
Orrell [24]	2010	Literature Review	Grade B Level 2	Step 2 Level 2
Nusrat et al. [10]	2019	Case Report	Grade C Level 4	Step 4 Level 4
Rezende Filho et al. [11]	2019	Prospective Cohort Study	Grade B Level 2	Step 3 Level 3
Ranjan et al. [12]	2019	Case Report	Grade C Level 4	Step 4 Level 4
Suzuki et al. [3]	2022	Prospective Cohort Study	Grade B Level 2	Step 3 Level 3
Pelletier et al. [13]	2013	Cohort	Grade B Level 2	Step 3 Level 3
Martin et al. [14]	2020	Prospective Cohort Study	Grade B Level 2	Step 3 Level 3
Juan et al. [15]	2020	Prospective Cohort Study	Grade B Level 2	Step 3 Level 3
Araki et al. [16]	2015	Case Report	Grade C Level 4	Step 4 Level 4
Pan et al. [17]	2016	Cohort Study	Grade B Level 2	Step 3 Level 3
McIlduff et al. [25]	2020	Diagnostic Study	Grade B Level 2	Step 3 Level 3
Tsunoda et al. [18]	2019	Retrospective Study	Grade C Level 4	Step 4 Level 4
Goyal and Mozaffar [19]	2015	Case Report	Grade C Level 4	Step 4 Level 4
Herath et al. [20]	2018	Case Report	Grade C Level 4	Step 4 Level 4
Van Den Engel-Hoek et al. [26]	2017	Literature Review	Grade B Level 2	Step 2 Level 2
Hagiwara et al. [21]	2021	Diagnostic Study	Grade C Level 4	Step 4 Level 4

Results

Selected Articles

**Figure 1:**
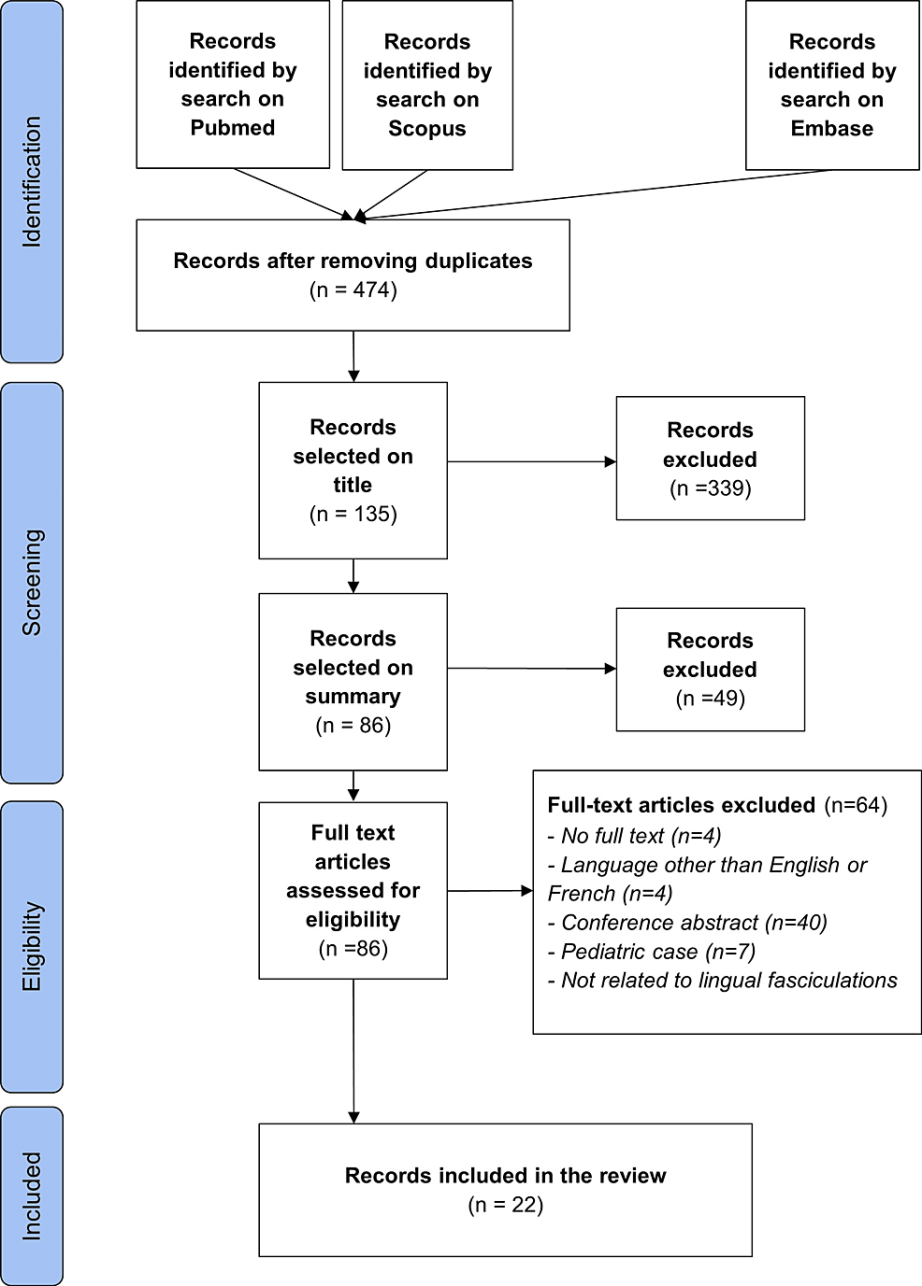
PRISMA flow diagram

Combining the data collected from the four database queries, we obtained 474 articles after removing duplicates (refer to Figure 1). Among these, 135 were deemed eligible for abstract screening, with 86 proceeding to full-text screening. Following the full-text screening process, 22 studies met the inclusion criteria for analysis, while 64 were excluded for various reasons, including unavailability of full text or lack of response from authors (n=4), non-English or non-French language (n=4), conference abstracts, movies, or letters (n=44), pediatric cases (n=7), and case reports lacking LFs (n=5), as illustrated in Figure 1.

Level of Evidence of the Articles

The analyses listed in Table 3 indicate the following results: 10 selected articles demonstrated a level of evidence graded as B according to the HAS and level 3 according to the Oxford score; two selected articles exhibited a level of evidence graded as B according to the HAS and level 2 according to the Oxford score; and 10 selected articles presented a level of evidence graded as C according to the HAS and level 4 according to the Oxford score.

Extraction of Clinical Data

In the clinical cases reported in Table 2, we identified eight distinct pathologies, including ALS, Brown-Van Vialetto-Van Laere syndrome, chronic inflammatory demyelinating polyradiculoneuritis (CIDP), Kennedy's disease, Machado-Joseph's disease (ASC3), poisoning by organophosphorus compounds, and alcoholism. LFs were observed in all of these conditions.

Epidemiology of Patients With LFs

**Figure 2:**
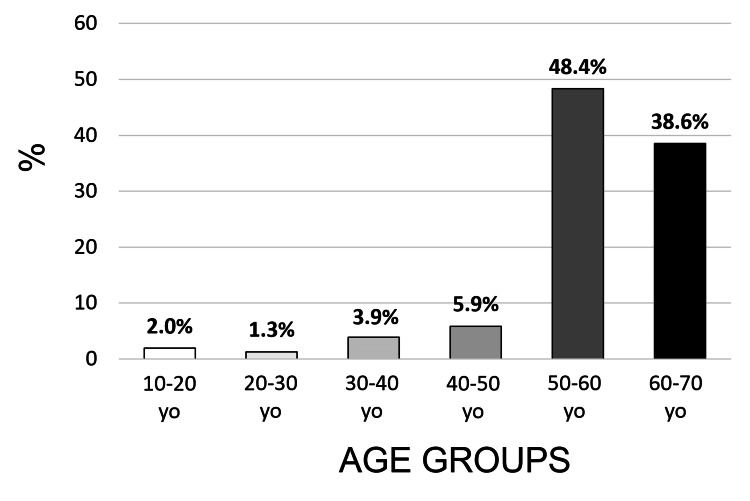
Epidemiological aspect Distribution of patients with LF (%) according to age (years). LF: lingual fasciculation; Yo: years old.

Our literature review identified 153 cases of patients with LF. Within this sample, the average age was 45.8 years, with the majority falling within the 50-60 and 60-70 age brackets (Figure 2). Women comprised 43% of the cases.

Pathologies Revealed by LFs

The majority of patients, 139 (91%), were diagnosed with ALS, seven with Machado-Joseph disease, two with familial amyloid transthyretin neuropathy, and one patient with other conditions. It can be concluded that in this study, nine out of 10 patients with LFs were diagnosed with ALS (Table 4).

**Table 4:** Pathologies causing lingual fasciculations listed in our study and the percentage of patients affected by each and treatment implemented according to the pathologies responsible for LF. LF: lingual fasciculation.

Diseases	n; %	Treatments used	
Amyotrophic lateral sclerosis	139; 91%	Riluzole® [1-3,29]	
Brown-Vialetto-Van Laere syndrome	1; 0.006%	Vitamin b2 (Riboflavin) [8]	
Chronic inflammatory demyelinating polyneuropathy	1; 0.006%	Methylprednisolone® and Rituximab® [9]	
Machado-Joseph disease	7; 0.046%	Symptomatic treatment [27]	
Bulbospinal amyotrophy or Kennedy's disease	1; 0.006%	Symptomatic treatment [28]	
Familial amyloid transthyretin neuropathy	2; 0.013%	Vyndaqel® [19]	
Osmotic demyelination syndrome	1; 0.006%	Sinemet® (treatment of extrapyramidal signs) [20]	

Methods Used for Detecting LFs

**Figure 3:**
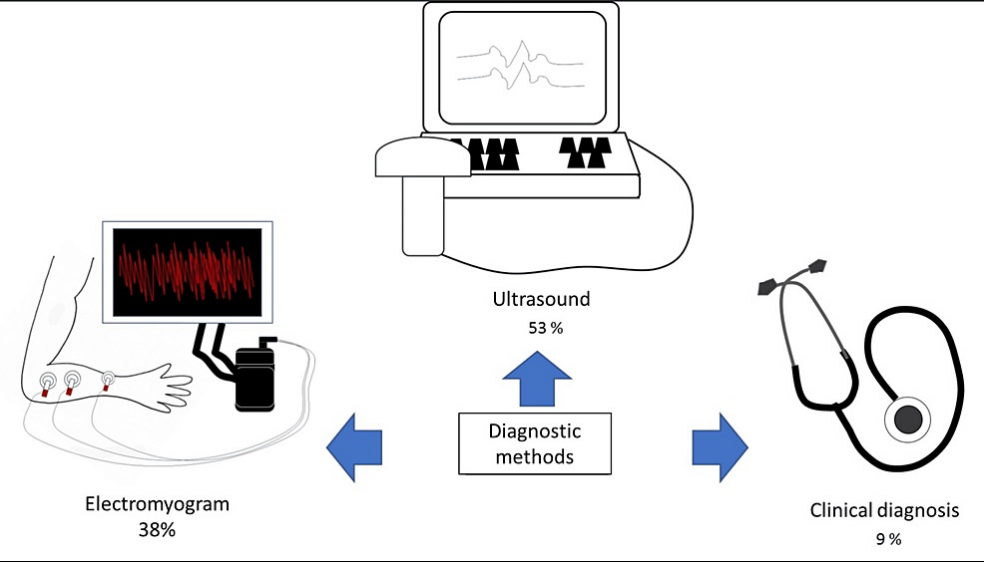
Exploring diagnostic approaches for lingual fasciculations: Utilization rates of various methods Source: Authors.

Among the articles we selected, 82 out of 153 cases, representing 53% of all listed patients, were diagnosed using ultrasound. Additionally, 58 cases were diagnosed using EMGs out of 153, accounting for 38% of all listed patients (Figure 3). The remaining cases were diagnosed through clinical observation methods, though the specifics were not described.

Consequences and Symptoms of LFs and Associated Pathologies

**Figure 4:**
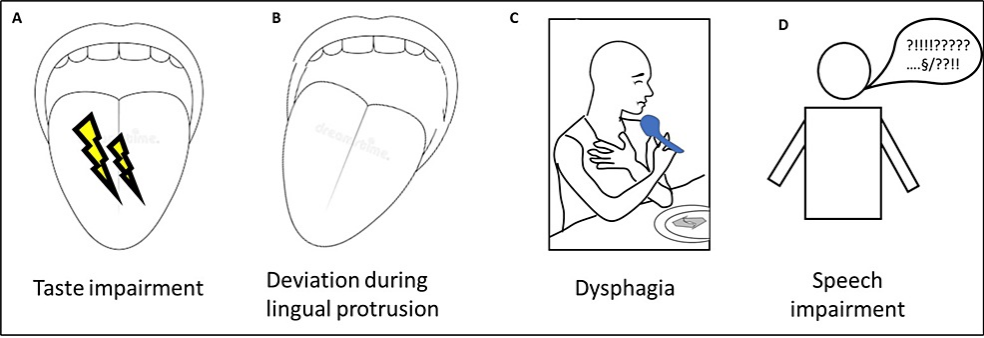
Consequences of lingual fasciculations (A) Taste alteration, (B) deviation during lingual protrusion, (C) dysphagia, (D) speech disorder. Source: Authors.

The selected articles shed light on various symptoms linked to LFs or the underlying pathologies they indicate (Figure 4).

For instance, some authors have noted taste alterations. In a comparative study comparing patients with ALS to a healthy control group, a significant decrease in taste perception was observed among ALS patients with LFs [13]. ALS patients exhibited notably lower taste intensities compared to age- and sex-matched healthy controls [F(1,30)=3.903, p=0.011]. Of the 16 ALS patients, nine (51% of the sample) reported taste changes, with seven of these nine patients (78% of the relevant sample) experiencing tongue fasciculations [13]. Another article illustrated functional impairments, particularly deviation during lingual protrusion in cases of osmotic demyelination syndrome [20]. In a study by Van Den Engel-Hoek [26], LFs were cited as a cause of dysphagia and swallowing difficulties in patients with neuromuscular disorders, leading to complications during the oral phase of swallowing.

Finally, one article reported dysphasia and speech disorders in four patients with LFs associated with ALS. Among eight suspected ALS cases, six received a definitive diagnosis of bulbar ALS based on neurological assessments. All six presented with slow, slurred speech, with four of them displaying LFs [18].

Treatments for Managing LFs

The management of these patients primarily revolves around addressing the underlying pathologies responsible for LFs (refer to Table 4).

In the case of ALS, Riluzole® (Riluzole, List I, a prescription drug with restrictions) is the sole medication identified to have a beneficial effect on survival, as demonstrated in a randomized, double-blind, placebo-controlled trial involving ALS patients [29]. However, while this treatment has shown efficacy in prolonging survival, no therapeutic impact on motor functions, respiratory function, muscle strength, or motor symptoms, including fasciculations, has been established. Riluzole® was utilized in the treatment of nine patients with ALS [29].

Regarding chronic inflammatory demyelinating polyneuropathy, treatments such as methylprednisolone® and Rituximab® 500 mg were administered initially, followed by IVIG at 2 g/kg over two days (Table 4). However, these treatments did not lead to a reduction or disappearance of LFs. Nevertheless, according to the authors, immunotherapy did halt neurological deterioration [9].

In a case involving LFs suspected to be of toxic origin, intravenous atropine was administered (IV bolus of 1 mg followed by three additional IV bolus doses of 2 mg, 4 mg, and 8 mg at 5-minute intervals, followed by atropine infusion of 1 mg) (Table 4) [12,30].

For Machado-Joseph disease and bulbospinal amyotrophy or Kennedy's disease, treatment remains solely symptomatic at present [27,28].

Evolution of LFs

In all documented cases of LFs stemming from neurodegenerative pathologies, patients did not achieve stable lingual functionality despite the administered therapeutics [9,19]. Notably, there are no reported instances of patients being cured of these pathologies in the literature. The only exception is the case of a patient experiencing LFs following toxic ingestion of organophosphate pesticides with suicidal intent, who exhibited symptom improvement after treatment [11].

Discussion

The objective of this study was to identify pathologies associated with LFs, thereby aiding practitioners, such as dentists, general practitioners, and ENT specialists, in directing patients to appropriate services.

We found that ALS was the primary cause of LFs (91%), followed by Machado-Joseph disease (0.046%) and familial amyloid transthyretin neuropathy (0.013%). Other reported cases included Brown-Vialetto-Van Laere syndrome, chronic inflammatory demyelinating polyneuropathy, bulbospinal amyotrophy (Kennedy's disease), osmotic demyelination syndrome, and organophosphorus poisoning, each affecting one patient. Additionally, we observed associated symptoms such as taste alterations, dysphagia, dysphasia, speech disorders, and lingual protrusion deviations. EMG and ultrasound were the most commonly used diagnostic methods, utilized in 91% of cases. Treatments were tailored to the underlying pathology, with medications like Rizulole®, methylprednisolone®, and Rituximab® prescribed accordingly. Interestingly, only patients with intoxication-induced LFs showed symptom improvement post-treatment; neurodegenerative pathology patients experienced worsening symptoms.

This description is pivotal for aiding practitioners in their diagnostic process and promptly directing patients. Delayed diagnosis is common in conditions like ALS [31], highlighting the importance of early detection to initiate treatment and enrollment in clinical trials.

EMG is the current standard for exploring LFs, although it is invasive, painful, and time-consuming. Ultrasound offers a precise and dynamic alternative, showing promise in detecting fasciculations. While EMG excels in identifying fibrillations, muscle ultrasound is more sensitive in detecting spontaneous muscle activity, questioning the necessity of EMG as a first-line diagnostic tool. Moreover, muscle ultrasound can reveal structural changes, detect involuntary movements, and provide dynamic imagery during swallowing attempts [25,26].

The clinical cases highlighted the symptoms accompanying LFs that aid in diagnosing underlying pathologies. These symptoms serve as crucial indicators for differential diagnosis. Indeed, ALS presents with muscle weakness, loss of fine motor skills, stiffness, respiratory difficulties, dysphagia, fatigue, and muscle atrophy [17,21]. Brown-Vialetto-Van Laere syndrome exhibits muscle weakness, loss of sensitivity, diaphragmatic paralysis, respiratory difficulties, hearing and vision loss, axonal neuropathy, and dysfunction of the anterior horn of the spinal cord [8]. Machado-Joseph disease (spinocerebellar ataxia type 3) manifests as ataxia, progressive external ophthalmoplegia, cerebellar dysfunction, muscle weakness, tremors, and abnormal eye movements [17]. Kennedy's disease (bulbospinal amyotrophy) showcases proximal and bulbar muscle atrophy, muscle weakness, extraoral muscle fasciculations, neuronal degeneration, and motor neuropathy [8]. Familial amyloid polyneuropathy presents with sensory and motor neuropathy, dysautonomia, cardiomyopathy, weight loss, digestive disorders, and heart disease [19]. CIDP demonstrates progressive muscle weakness, altered sensation, reduced reflexes, hyperproteinorachia, demyelinating neuropathy, and potential clinical remissions [9]. Osmotic demyelination syndrome results in paralysis, altered consciousness, dysarthria, dysphagia, abnormal movements, cognitive disturbances, and cerebellar symptoms [20]. Finally, organophosphate poisonings exhibit muscle weakness, nausea, vomiting, excessive salivation, sweating, pupil changes, convulsions, and coma [11].

In summary, differential diagnosis relies on medical history, clinical evaluation, and supplementary tests such as radiography (EMG, MRI, electroneuromyography [ENMG]), genetic analysis, and biological assays. Future research should prioritize standardized methodologies and treatment efficacy across various pathologies.

This study has several biases. The clinical and histological descriptions of the pathologies were dependent on the practitioners, and only the information provided by the authors was considered. However, we ensured that the samples in the included studies were independent, meaning there was no overlap between the clinical cases presented. Since these were solely clinical case reports, this analysis did not require counting or analyzing the degree of variation in distributions. Moreover, the review encompassed various conditions associated with LFs, offering a comprehensive overview of their etiologies.

Future studies should focus on improving data related to this pathology. First, investigators should strive for methodological consistency to facilitate comparability and synthesis of results across studies, addressing one of the biases in our work. Additionally, further clinical research is warranted to explore the efficacy of different treatments for LFs across all underlying pathologies, including long-term outcomes.

## Conclusions

In conclusion, this study examined a total of 22 studies that met the inclusion criteria. We identified eight distinct pathologies associated with LFs, with ALS representing the majority of cases. The results also highlighted the methods used to detect LFs, as well as the symptoms and treatments associated with different pathologies. It is noteworthy that despite the treatments administered, patients did not achieve stable lingual functionality in cases of neurodegenerative pathologies. This work emphasizes the need for more homogeneous research methodologies to facilitate comparability of results across studies and provides an overview of the clinical and diagnostic aspects of LFs. Practitioners must be able to detect LFs, know their etiologies, and promptly refer the patient to appropriate specialized services, allowing for early integration into a care protocol and attempts to improve prognosis.
